# Non-protein coding RNA biomarkers and differential expression in cancers: a review

**DOI:** 10.1186/1756-9966-27-19

**Published:** 2008-07-16

**Authors:** Massimo Mallardo, Palmiro Poltronieri, Oscar Fernando D'Urso

**Affiliations:** 1 University of Napoli Federico II, Department of Biochemistry and Medical Biotechnologies, Via S. Pansini 5, Napoli, Italy; 2 National Research Council, Institute of Sciences of Food Productions, Ecotekne, via Lecce-Monteroni, Lecce, Italy; 3 Biotecgen, Ecotekne, via prov.le Monteroni, Lecce, Italy

## Abstract

**Background:**

In these years a huge number of human transcripts has been found that do not code for proteins, named non-protein coding RNAs. In most cases, small (miRNAs, snoRNAs) and long RNAs (antisense RNA, dsRNA, and long RNA species) have many roles, functioning as regulators of other mRNAs, at transcriptional and post-transcriptional level, and controlling protein ubiquitination and degradation. Various species of npcRNAs have been found differentially expressed in different types of cancer. This review discusses the published data and new results on the expression of a subset of npcRNAs.

**Conclusion:**

These results underscore the complexity of the RNA world and provide further evidence on the involvement of functional RNAs in cancer cell growth control.

## Background

In recent years a large number of non-protein coding transcripts has been discovered. Many transcripts have been identified with low coding potential, possessing only short open reading frames (smaller than 200 nucleotides), potentially coding for 50–70 amino acid small peptides. The possibility of coding for peptides has been excluded for npcRNA in the following cases: absence of ORFs longer than 60 bases; presence of ORFs but absence of Kozak sequences at translation start sites; or no interspecies phylogenetic conservation of the first methionine codon, or absence of conservation in the ORF in related species analysed by comparative genomic analysis. Bioinformatic methods can assist in the discrimination of non-coding from coding sequences, based on four principles: open reading frame size, sequence similarity to known proteins or protein domains, statistical models of protein-coding sequence, and synonymous versus non-synonymous substitution rates [1].

Some npcRNAs affect transcription [2] and chromosome structure [3]. The human 7SK RNA binds and inhibits the transcription elongation factor P-TEFb. 7SK RNA and the components of the splicing apparatus U1–U6 snRNAs are implicated in the regulation of transcriptional elongation. TFIIH (transcription factor IIH), a general transcription initiation factor, appears to associate specifically with U1 snRNA, a core splicing component. A human npcRNA, the steroid receptor activator (SRA) RNA, was identified as interacting with progestin steroid hormone receptor and may serve as a coactivator of transcription [4,5]. In SRA, the production of a peptide is an additional function. Several extremely long npcRNAs detected in mammalian cells have been implicated in silencing genes and changing chromatin structure across large chromosomal regions. Examples include the human *Xist *RNA required for X chromosome inactivation and mouse *Air *RNA required for autosomal gene imprinting. The chromosome-associated RNA has been proposed to recruit proteins that affect chromatin structure, in order to establish and/or maintain gene silencing. An eukaryote-specific RNA that is required for proper chromosome replication and structure is the telomerase RNA. This ncRNA is an integral part of the telomerase enzyme and serves as the template for the synthesis of the chromosome ends. Basal telomerase activity is dependent on expression of the hTERT and hTR genes and upregulation of telomerase gene expression is associated with tumour development, and features of the tumour environment itself may influence telomerase gene regulation. The majority of solid tumours contain regions of hypoxia: it has recently been demonstrated that hypoxia can increase telomerase activity. The chromatin landscape of the telomerase promoters during hypoxia, revealed dynamic recruitment of a transcriptional complex involving the hypoxia-inducible factor-1 transcription factor, together with p300, RNA polymerase II and TFIIB. HIF-1 traffics along and remains associated with the hTERT gene as transcription proceeds. This study showed that hTERT and hTR are subject to similar controls under hypoxia highlighting the rapid and dynamic regulation of the telomerase genes in vivo [6]. Also in the analysis of the function of Vault complex (formed by the Vault protein, V-PARP and Vault-RNA) by using mouse knocked out in each Vault-component, some of these components has been associated to cellular functions as: intracellular trafficking, lipid rafts, and cell proliferation (E. Wiemer, personal communication).

RNAs also regulate mRNA stability and translation. Some npcRNA mimics the structures of other nucleic acids; the 6S RNA structure is reminiscent of an open bacterial promoter, and the tmRNA has features of both tRNAs and mRNAs. Other npcRNAs, such as the RNase P RNA, have catalytic functions. Although synthetic RNAs have been selected to have a variety of biochemical functions, the number of natural npcRNAs shown to have catalytic function is limited. Many npcRNAs are associated with proteins and augment their functions. One example is the RNA activators of dsRNA-protein kinase [7]. The heat-shock RNA, Hsr-1, switches on the transcriptional activity of heat shock factor HSF-1 by inducing trimerization. Other npcRNAs, such as the snRNAs and the SRP RNA, serve key structural roles in RNA-protein complexes. Expression studies helped in correlating a specific induction factor and the expression of transcripts with unknown function. As example, the oxygen radical response induces AK001558, a npcRNA often overexpressed in cancers. AK027352 is a gene regulated by hypoxia, often overexpressed in cancers, with features of transmembrane proteins but also of npcRNAs as well.

It has become clear, also from chromosome tiling experiments using DNA microarrays, that most part of the genome is transcribed, also in genomic regions previously thought to contain junk DNA, and that many introns give rise to new RNA species. In fact, not only these small RNA species are constituents of RNP particles, but in many cases regulate the alternative splicing of essential proteins, a phenomenon that has great implication in inflammation, in disease and in cancer.

The H-Invitational (H-Inv) database of human full length cDNAs, update 3.4 released in april 2006, contain about 30000 protein coding genes, 15000 peptide coding transcripts, a set of transcribed pseudogenes, and a huge array of putative genes and candidate npcRNAs lacking any open reading frame [8]. In addition to these genes, it was shown that 10% of the protein coding genes are cotranslated with its antisense RNAs that regulate the levels of expression of the sense mRNAs.

Nowadays, the most comprehensive database of npcRNAs is curated by the ARC Research Centre in Brisbane [9] with annotation of human, mouse, and literature curated ncRNAs, an overview to the PubMed data, a BLAST function, and functional data and tissue expression patterns. Human and mouse orthologs were screened for sequence conservation and software for analysing secondary structures such as QRNA, RNAfold, RNAz [10], Evofold [11] and Foldalign [12]. Three-dimensional architectural motifs are increasingly recognized as determinants of RNA functionality [13]. Such motifs can encode spatial information. RNAs are targeted to subcellular localities in many eukaryotic cell types, and especially in neuronal and glial cells, RNAs can be transported over long distances to their final destination sites. Such RNAs contain cis-acting long-range targeting elements, and recent evidence suggests that kink-turn motifs within such elements may act as spatial codes to direct transport. Kink-turns are complex RNA motifs that feature double- and single-stranded components and introduce a signature three-dimensional structure into helical stems.

Recently, the Encyclopedia Of DNA Elements (ENCODE) Project was set up in order to identify all functional elements in the human genome. The pilot phase is for comparison of existing methods and for the development of new methods to rigorously analyze a defined 1% of the human genome sequence. Experimental datasets are focused on the origin of replication, DNase I hypersensitivity, chromatin immunoprecipitation, promoter function, gene structure, pseudogenes, non-protein-coding RNAs, transcribed RNAs, multiple sequence alignment and evolutionarily constrained elements. These databases links to the University of California Santa Cruz browser, to visualise the genes on the chromosomes [14].

A recent paper [15], in which long SAGE was used to identify 660000 transcripts isolated from a human brain, described more than 20% novel and differently expressed transcripts, showing that the actual number of genes in human genome contain probably twice the number of genes and transcribed regions.

The Head and Neck Annotation Consortium, as well as the ENCODE consortium, produced a huge number of transcripts of unknown function, often originating from introns and from untranslated regions of the genome [16]. It is hypothesised that some of them functions as npcRNA.

The majority of the well-known ncRNAs are relatively abundant. It was shown that long RNAs can produce small RNA species, as in the case of H19 that give rise to small RNA as well as to one identified microRNA [17]. The 130 kb deleted region on chromosome 3p12 in lung (GLC20 line) and breast (U2020 and HCC38) cancer cell lines was found to contain, near the protein coding gene ROBO1/DUTT1 (Deleted in U2020), two npcRNAs with short segments highly conserved among mammal genomes, that are highly overexpressed in various cancers in respect to normal tissues [18].

In a functional analysis of npcRNAs through high-throughput screens, a repressor of NFAT was found [19]. This npcRNA, NRON, by interacting with β-importins, negatively regulates the nuclear trafficking of NFAT. In another case, the expression of the dihydrofolate reductase (DHFR) gene is repressed by one of its regulatory transcripts produced from the minor promoter showing a mechanism of promoter-specific transcriptional repression [20]. EGO was shown to be essential for the expression of major basic protein in eosinophils [21]. However, while a group of npcRNAs is conserved between mammalian genomes, a second group has been recognized as primate-specific [22] by applying phylogenetic and comparative genomics analyses.

The mechanisms of action for the characterized ncRNAs can be grouped into several general categories. There are ncRNAs where base-pairing with another RNA or DNA molecule is central to function. Antisense RNAs can regulated the induction of transcripts from different promoters or producing a spectrum of UTRs with various lengths. One recent finding showed the regulation of the PTEN induced putative kinase 1 (PINK1) locus by a natural antisense producing a dsRNA [23]. There are snoRNAs that direct RNA modification, RNAs that modulate translation by forming base pairs with specific target mRNAs, and probably most of the miRNAs are examples of this category. Sno-like RNAs and other classes of regulatory RNAs originate either from introns and exons of protein coding and npcRNA genes produced by the RNA Polymerase II transcription machinery, or transcribed (as H1, U6 or 7SK) by the RNA Pol III machinery. Recently, new human ncRNAs have been detected and functionally characterised, which have high homology with protein-coding genes [24]. The authors' data confirms the existence of a sense/antisense-based gene regulation network, where part of the Pol III transcriptome could control its Pol II counterpart. In particular, the 21A transcript was shown to co-regulate CENP-F gene and to modulate CENP-F expression at post-transcriptional level: 21A overexpression specifically inhibited cell proliferation in their culture system [24]. This effect was related also to the finding that 21A ncRNA is downregulated in tumour cell lines (HeLa, 293T, and LAN5).

The microRNAs is the class of 21 base-long small RNAs, having a hairpin structure, that affect target genes by forming perfect matches or base pairing to highly similar sequences. In sheep, mutant Texel MSTN mRNA has mistakenly become the target of miRNAs because of its disguise using a target octamer motif borrowed from genuine target genes. This phenomenon led to muscular hypertrophy in the "callipyge" sheep. In analogy, other genes were found deregulated, as PEG11 and its antisense transcript [25]. Subject to a similar mechanism of regulation, many human disease susceptibility loci are related to SNPs within a microRNA or its target gene.

## Cancers

It has recently become evident that cancer is also subject to translational control [26]. Several cancer-related regulatory events are mediated by the cap-dependent mRNA binding stage of translation initiation. This implicates that cap-dependent protein synthesis pathway is a pleotropic integrator and amplifier of many essential oncogenic signals, and the translational control network as a bona fide molecular target for regulation by RNA sequences. The most important changes leading to cancer development are caused by DNA mutations, deletions and chromosomal rearrangements. Besides genotypic alterations, epigenetic events as DNA hypermethylation or post-translational modification of histones and DNA binding proteins mostly affect the physiological levels of expressed proteins (phenotypic alterations). Different regulation and usage of promoters and alternative splicing produce new protein isoforms. Alternative spliced isoforms of anti-oncogenes (devoid of activity) and constitutively-activated spliced forms of signalling proteins are cancerogenic, and upregulation of the splicing factor SF2/ASF transforms immortal rodent fibroblasts [27].

Although reprogramming of chromatin normally has been shown during early telophase, the effect of single-stranded (ss) and double-stranded (ds) RNAs (activator RNA, fountain RNA, antisense RNA) can result in chromatin reprogramming during interphase. The formation of by base-pairing between complementary RNAs may elicit regulatory responses at the transcriptional level. Analysis of antisense transcription at several imprinted loci has suggested a number of other mechanisms that may not require formation of dsRNA [28]. Many molecular lesions after transformation are DNA-based, including chromosomal amplification, deletion, mutation, and translocation. Epigenetic lesions, not involving directly DNA sequences, are RNA-based, and perhaps amenable to RNA exchange during reprogramming. An interesting experiment [29] published in 1963, showed that pure isolated RNA from normal bone marrow was able to temporarily reverse into a normal phenotype the neoplastic state of human acute myelocytic leukemia cells following intra-marrow injection. RNA-based lesions may be more reversible than DNA-based lesions within human neoplasms. Many cellular pathways are implicated in cancer development, as the DNA damage response, the growth arrest, the regulation of survival genes, and the apoptotic pathway. For some microRNA, a direct role in some of these pathways has been shown. In the case of the growth arrest genes, gas5, which contains intronic sequences producing small nucleolar RNAs, is itself a npcRNA [30].

### npcRNAs implicated in cancer

Cancer is a multistep genetic and epigenetic disease with a complex etiology. Several defects such as mutations, down-regulation, over-expression and deletions in oncogenes and tumor suppressor protein-coding genes have been extensively described in cancer cells. Recently, transcriptome analysis and different experimental approaches comparing tumor cells to normal ones are providing strong evidence that defects in ncRNAs might occur in tumors [31,32]. The H19 gene was the first example of a large ncRNA associated with tumors to be described [33] and we are now facing a growing list of ncRNA transcripts implicated in different types of cancer [34]. Some of these examples are discussed in the next sections and shown in Table 1. The metastasis-associated lung adenocarcinoma transcript 1 (MALAT-1) gene is one of the major genes upregulated in Endometrial stromal sarcoma (ESS) of the uterus a rare uterine malignancy. In addition, MALAT-1 is found overexpressed in other cancers as in endometrial sarcoma of the uterus [35] and in hepatocellular carcinomas. It was recently identified as noncoding nuclear enriched abundant transcript (NEAT2) from the locus on chromosome 11 coding for two TncRNAs, and is the polyadenylated component of nuclear speckles [36]. NAMA, found downregulated in papillary thyroid carcinomas [37], is a gene induced by the DNA damage response. OCC1 is overexpressed in colon carcinomas [38]. TRNG10 has been related to various types of cancer [39]. His-1, a gene involved in leukemias by frequent viral insertion in mouse, has a human counterpart [40]. HOST2 is a npcRNA gene expressed in ovarian cancer cells [41]. Ovarian cancers show alteration of the 17q25 region, in which the SEPT9 locus originates a large number of transcripts. A difference in the level of the produced mRNAs, that encode exactly the same polypeptide, is present in their 5'UTR. It is postulated that variations in the use of the untranscribed regions may affect the production and amount of proteins. The down-regulation of the cytoglobin gene, in tylosis with oesophageal cancer was shown to be caused by a trans-allele repression mechanism [42]. Many of the mechanisms that modify the UTRs, or that affect differential splicing, make use of RNA regulation, antisense RNAs, and may involve RNP complexes.

**Table 1:** Examples of ncRNAs implicated in cancer (from Prasanth and Spector 2007, with added RNA species

**Transcript**	**Organism**	**Size ** **(nt)**	**Genome ** **map**	**Deregulation**	**Type of cancer**	**References**
Deleted in U2020	human		3p12	Overexpression	Lung, breast, other	[18]
NCRSM	human		12q23.1	Overexpression	rhabdomyosarcoma	[95]
HIS-1	Mouse	3000	2	Overexpression	Myeloid leukemia	[40]
DD3	Human	2000–4300	9q21-22	Overexpression	Prostate	[96]
PCGEM1	Human	1603	2q32	Overexpression	Prostate	[38]
BC200	Human	200	2p16	Overexpression	Breast, parotid, cervix, esophagus, lung, ovary, tongue	[97]
MALAT-1	Human, mouse	8000	11q13	Overexpression	NSCLC	[98,98]
H19	Human	2700	11p15.5	Loss of imprinting, overexpression	Liver and breast	[33]
21A	Human		CENP-F Antisense	Down-regulation	293T, HeLa	[24]
miR-143	Human	22	5	Down-regulation	Colon	[100]
miR-145	Human	22	5	Down-regulation	Colon	[100]
miR-155/BIC	Human	22	21q21	Overexpression	Burkitt and B cell lymphomas	[71]
miR-15a	Human	22	13q14	Deletion and/or downregulation	B-CLL	[64]
miR-16a	Human	22	13q14	Deletion and/or downregulated	B-CLL	[64]
let-7	Human	21–25	22	Downregulated	Lung adenocarcinoma	[68]
NAMA	Human	873	9q22.33	Downregulated	Papillary thyroid carcinoma	[37]

### Hormone dependent cancers

The function of some npcRNA as tumor suppressor and of other as oncogenes is not well understood, but in many case the resistance to apoptosis or the proliferative ability do not well relate with tumour development. Mammary gland development is associated to the induced expression of the npcRNA PINC [43], in cells that have progenitor-like ability but show inhibition of carcinogen-induced proliferation. Breast cancer with oestrogen receptor positivity is associated with high expression of ncRNAs as SRA [5,44]. The up-regulation of SRA in many human tumours of steroid-dependent tissue may reflect a cellular effort to antagonize excessive proliferation. Additional studies are needed to elucidate the mechanisms involved. It is possible that tumor progression is controlled by the specific composition of ribonucleoprotein complexes containing SRA, whose expression level determines whether transcriptional coactivators or corepressors are incorporated. This model is consistent with to the reported role of SRA in attenuation of Steroid Receptor transactivation. Even so, the analysis of SRA in mouse models suggest that the promotion of proliferating functions used to achieve established tissue structures occurs in conjunction with mechanisms to prevent tumour formation. H19 is found at high levels in the cell line SKBR3 overexpressing the ERBB2 kinase; H19 is known to produce also a microRNA [16]. Duplex RNAs complementary to the progesterone receptor (PR) promoter increased the expression of PR protein and RNA after transfection into cultured T47D or MCF7 human breast cancer cells [45]. Upregulation of PR protein reduced the expression of downstream cyclooxygenase 2 gene but did not change concentrations of estrogen receptor, demonstrating that activation of RNAs can manipulate physiologically relevant cellular pathways. Prostate cancer is an hormone-dependent tumour in which androgen receptor activity is related to the expression of PSA (prostate specific antigen) as well as to other cancer-associated npcRNAs (DD3, PCGEM1). In some cases, sensitivity to low levels of androgen is increased by amplification, mutations and/or elevated levels or broadened specificity of co-activators of the androgen receptors. After anti-androgenic treatment, primary prostate cancers can also shift to an androgen-independent state and become recurrent, activation of androgen receptors occurring in the absence of androgens due to crosstalk via other signalling pathways. A correlation was shown between androgen receptor activation and the expression of intronic npcRNAs [46], in the antisense direction in the majority of cases, in LNCaP cells treated with the hormone. In addition, the binding of the nuclear receptor to an upstream regulating region of an intron was demonstrated.

#### Resistance to chemotherapeutics and malignant phenotype

Many chemotherapeutics used for cancer treatment may be ineffective due to the expression of npcRNAs. It was shown that the overexpression of PCGEM1, a prostate associated npcRNA, inhibited the apoptosis induced by doxorubicin, etoposide and sodium selenite, in the LNCaP cell culture model [47]. The attenuation of apoptotic response was shown to be androgen dependent, ass is the induction of PCGEM1 expression. A major obstacle for clinicians in the treatment of advanced prostate cancer is the inevitable progression to chemo-resistance, especially to docetaxel and taxans. It was shown that this effect is mediated through the activation of STAT1 phosphorylation, and downstream to the expression of clusterin. It would be important to individuate the mechanisms involving proteins and possibly npcRNAs that could block this pathway. Many data are known on the beneficial activity of curcumin, tea polyphenols and other flavonoids on the inhibition of STAT1 activation. Resveratrol was shown to increase the deacetylase activity of sirtuins. It is not yet known the identity of possible partners in the control of this pathway and the cellular components inside the cell on which bioactive plant compounds exert their effect. It could be possible that some npcRNA can bind to bioactive compounds and be regulated by a riboswitch-like mechanism.

#### Leukemias

Deleted in leukemia (Leu-1), a npcRNA gene transcribed head to head to Leu-2 in the opposite direction, is located on chromosome 13q14, in the 30-kb region of loss in chronic B-cell lymphocytic leukaemia, near the miR-15/miR16 cluster [48]. The homozygous loss of this region has great effects on the regulation and control of normal CD5+ B lymphocytes and their homeostasis. Leu-1 was found expressed in normal thymus, testis, intestine, and to a lesser extent in other tissues. BCMS was found to be a long mRNA containing Leu-1 and the most likely candidate for the tumor suppressor gene in 13q14 [49]. In this review, we will not discuss the cases of snoRNAs found in the chromosomal breakpoints involved in some types of human B-cell lymphoma and other leukemias, since their location in the breakpoints could be a mere coincidence. HIS-1 is a common site of viral insertion in mouse. His-1, when overexpressed, induces myeloid leukemias. The 5'-flanking region is highly conserved in the human homolog. Sequence comparisons between the mouse and human genes identified conservation of other putative functional domains in exon 3 and in each of the two introns, but none of the multiple candidate ORFs in the mouse RNA were conserved in the human sequence, suggesting that the RNA may be the final and functional product from the human and mouse His-1 genes [40].

#### microRNAs in cancers and leukemias

Few classes of ncRNAs have a well-defined function. One group that is well characterized, at the biochemical level, is represented by miRNAs. This group comprises a large class of small non coding RNAs (~22-nucleotide RNAs) acting through base pairing to partially complementary sites in the 3' untranslated regions (3'UTR) of the targeted messenger RNA [50]. This lead to reduced translation of the related proteins (natural RNAi) [51]. Similarly to transcription factors, miRNAs can play an essential role in regulation of gene expression and control of the cellular fate. miRNAs biogenesis has very peculiar features if compared to other ncRNAs classes, such as two maturation steps occurring in different subcellular districts. Several miRNAs are embedded within introns of protein-coding genes or non-coding RNA transcripts [52]. This observation suggests that a large number of miRNAs might be transcriptionally linked to the expression of their host-gene promoters. An amplification or a block of the effects of miRNAs on their target genes is observed when nucleotide substitutions appear in microRNAs or in its target genes, blocking thus their interaction. miRNAs are generated as a primary transcript (pri-miRNA) by RNA polymerase II [53] or by RNA polymerase III-like viral miRNAs [54]. Pri-miRNAs are capped, polyadenylated [55] and subsequently they enter a microprocessor complex (500–650 kDa) consisting of a Drosha (an RNase III endonuclease) and an essential cofactor DGCR8/Pasha protein containing two double-stranded RNA binding domains [56,57]. There, they are processed and cleaved giving rise to another precursor of 60–80 nucleotide stem-loop sequence (pre-miRNA) with a 5' phosphate and two 3' nucleotide overhang. The pre-miRNAs are then transported to the cytoplasm by Exportin-5, a member of the Ran transport receptor family [58]. Finally, the stem-loop structure is sequentially processed by the cytosolic RNase III Dicer [59] to yield the mature single-stranded miRNA. The single-stranded mature miRNA is incorporated into the cytosolic effector complex, called RNA-Induced Silencing Complex (RISC). Within the RISCs miRNA exarches its function of translational silencing by base-pairing the 3' UTR of the specifically targeted mRNAs [60,61].

Recently, it has been shown that in addition to this mechanism, silencing by miRNA can be triggered by a miRNA-induced rapid deadenylation of the messenger RNA that leads to rapid degradation of the latter [62].

Although the elucidation of miRNAs function has not progressed as other aspects in the field, the role of this family of ncRNAs in the molecular aetiology of cancer has been extensively investigated. Several laboratories have modified DNA microarray technology to form miRNA microarray technology. Using this technique they have performed the miRNA expression profile (miRNome) in cancer patients with solid tumors or hematological malignancies and found that miRNAs are differentially expressed in normal and tumor tissues [63]. These differences are often tumor-specific and, in some cases, can be related to prognosis.

One of the firsts evidences for the involvement of miRNAs in cancers came from molecular studies characterizing the 13q14 deletion in human chronic lymphocytic leukemia (CLL) and the localization of human miR-15a/miR-16 cluster and miR-146 at the chromosomal breakpoint or deletion sites [48]. These data suggested their possible involvement in the aetiology of the chronic lymphocytic leukemia [64]. (Calin et al. 2002) and multiple myeloma [65]. Further, Cimmino and colleagues [66] showed that miR-15 and miR-16 induce apoptosis by targeting antiapoptotic gene B Cell Lymphoma 2 (BCL2) mRNA, a key player in many types of human cancers, including leukemias, lymphomas, and carcinomas [67]. In this respect, miR-15 and miR-16 can be considered as potential tumor suppressor genes. Another miRNA, Let7a, could be a potential tumor suppressor gene. Takamizawa [68] showed that let7a was significantly downregulated in lung cancer. This data was confirmed by the finding that let7a overexpression in lung cancer cell lines inhibited cell growth *in vitro*. Moreover, Johnson et al. [69] demonstrated that Ras protooncogene mRNA is targeted by let7a and this leads to repression of Ras protein translation. Lung tumor tissue displays low let7a expression whereas Ras protein is significantly overexpressed compared to normal lung tissue [68]. Conversely, miR155, encoded by the non-protein coding Bic gene, displays numerous oncogenic features. Recently, Costineau [70] showed that selective overexpression of miR155 in B cell, constitutively driven by Eμ-enhanced promoter, led to B-cell polyclonal proliferation followed by high-grade lymphoma pre-B leukemia. In accordance to this observation, miR155 is overexpressed in pediatric EBV-positive Burkitt lymphoma [71], Hodgkin lymphomas, primary mediastinal and diffuse large B-cell lymphomas [72], and in EBV-immortalized B-cell lines (Mallardo et al., unpublished results). Although the above mentioned works seems to answer the question of whether miRNAs can act as oncogenes or tumor suppressor genes, further work is needed to fulfil this important issue. Indeed, a peculiar situation is represented by miR-17-92 cluster. He and colleagues [73] showed that overexpression of miR17-92 cluster obtained by viral transduction in hematopoietic stem cells from Myc transgenic mice strongly triggers tumor development. This finding can indicate an oncogenic function of the miR cluster. Consistently, overexpression of miR-17-92 cluster has been observed in human lung cancer where it enhances cell proliferation [74]. On the other hand, O'Donnell found that overexpression miR-17-92 cluster, directly driven by c-myc-binding to the cluster promoter, provokes downregulation of E2F1 translation whose mRNA is targeted by miR17 [75]. E2F1, a myc target, promotes cell proliferation. According to these findings miR-17-92 cluster would act as tumour suppressor gene.

If it is currently accepted that miRNAs can play a central role in the molecular aetiology and maintenance of cancer, several groups are investigating to address the question of whether the miRNA expression profile could become a useful biomarker for cancer diagnosis and prognosis. Data on miRNA expression profile, obtained by miRNA microarray, on cohorts of patients with cancer compared to normal people are giving raise evidences that miRNA are differentially expressed in cancer and normal tissue. The evaluation of miRNA expression profiles of 10 normal and 76 neoplastic breast tissues, using miRNA microarray, showed that the miRNAs expression patterns were significantly different between normal and neoplastic breast tissues [76]. In particular, miR-125b, miR-145, miR-21, and miR-155 were significantly reduced in breast cancer tissues. Furthermore they observed an interesting correlation between the expression of miRNAs and specific breast cancer biopathologic features, such as tumour stage, proliferation index, oestrogen and progesterone receptor expression, and vascular invasion [76]. Another report shows the correlation of high miR-155 expression with short survival in lung cancer pointing out the importance of miRNA expression pattern with prognosis [64]. A large miRnome analysis on 540 samples including breast, lung, stomach, prostate, colon and pancreatic tumours identified a solid-cancer signature of overexpressed miRNAs, such as miR-155, miR-17-5p, miR-20a, miR-21, miR-92 and miR-106a [77]. Calin [64] reported significant differences in miRNA expression between human Chronic Lymphocytic Leukemia B cells and their normal counterpart CD5^+ ^B cells. Lately, it has been shown a link between a miRNA signature and prognosis and progression of CLL [65].

Although miRNAs are a newly discovered class of ncRNAs, they have quickly got importance in molecular oncology. Therefore, it seems to be clear that the understanding of their function and the investigation of the expression profile of these peculiar ncRNAs will provide insight in molecular basis of cancer and, in the near future, powerful tools for diagnosis, prognosis and classification of cancers.

### Tumour-associated viruses and npcRNAs

Globally, it is estimated that 20% of all cancers are linked to infectious agents. Studies of oncogenic DNA viruses have contributed to the understanding of key molecular mechanisms of tumorigenesis and viral oncogenicity. Human T-cell leukaemia virus type 1 (HTLV-1), a retrovirus that infects 20 million people worldwide, was the first retrovirus to be shown to be causal for a human cancer, adult T-cell leukaemia (ATL). The maintenance of ATL transformation seems to require the function of a novel antisense protein and RNA, termed HTLV-1 basic leucine zipper factor (HBZ). A mechanism by which viruses survive inside cells is by inactivating the cellular antiviral machinery, or inactivating the RNA interference response, or acting on the dsRNA-activated protein kinase (PKR). Virally encoded oncoproteins such as adenovirus E1A and human papillomavirus (HPV) E7 can bind an array of cellular proteins to override proliferation arrest. Adenovirus VA1 noncoding RNA can inhibit small interfering RNA and MicroRNA biogenesis, both by inhibiting nuclear export of shRNA or premicro-RNA precursors, competing for the Exportin 5 nuclear export factor, and inhibiting Dicer function by direct binding of Dicer [78]. Recently, many viral-encoded miRNAs have been discovered, mostly in viruses transcribed from double-stranded DNA genomes. Several virus-encoded miRNAs have unique aspects to their biogenesis, such as the polymerase that transcribes them or their location within the precursor transcript. Additionally, viral interactions with cellular miRNAs have also been identified, and these have substantially expanded the knowledge of miRNA functions. The functions of most viral-derived miRNAs are unknown; however, functions have been documented or proposed for viral miRNAs from three different viral families-herpesviruses, polyomaviruses, and retroviruses.

HPV and HSV are the most well known viruses that act through the production or through the regulation of cellular npcRNAs. The family of human herpesvirus include pathogens such as herpex simplex virus, Epstein-Barr virus (EBV), human cytomegalovirus (HCMV), Kaposi's sarcoma herpesvirus (KSHV), and for murine tumours the herpesvirus 68 (MHV68). These viruses share the ability to establish latency in the host after an early replication phase. The Epstein-Barr virus (EBV) is a human herpesvirus that is normally carried lifelong as an asymptomatic infection. However, EBV is also the causative agent of infectious mononucleosis and is linked to the development of several malignant tumours, including B-cell neoplasms such as Burkitt's lymphoma and Hodgkin's disease, certain forms of T-cell lymphoma, and epithelial and nasopharyngeal cancers. Epstein-Barr virus (EBV) is shown to express at least 17 distinct microRNAs (miRNAs) in latently infected cells, which potentially regulate both viral and cellular genes. The BART miRNAs are expressed at high levels in latently infected epithelial cells and at lower, albeit detectable, levels in B cells. The BHRF1 miRNAs are found at high levels in B cells undergoing stage III latency but are essentially undetectable in B cells or epithelial cells undergoing stage I or II latency. Induction of lytic EBV replication was found to enhance the expression of many, but not all, of these viral miRNAs. In addition, oncogenic potential of EBV, independent of its effect on c-myc-induced apoptosis, is exerted by 2 highly expressed small non-protein-coding RNAs, EBER1 and EBER2. In Herpesviruses, EBER RNA block the activity of dsRNA-PKR in infected cells.

The pathogenic Kaposi's sarcoma-associated herpesvirus (KSHV) encodes an array of 12 distinct miRNAs [79] all of which are expressed at readily detectable levels in latently KSHV infected cells. Target candidate genes of the viral microRNAs include several mRNAs previously shown to be down-regulated in KSHV-infected cells. Herpesvirus: The latency-associated transcript (LAT) of herpes simplex virus-1 (HSV-1) is the only viral gene expressed during latent infection in neurons. LAT inhibits apoptosis and maintains latency by promoting the survival of infected neurons. No protein product has been attributed to the LAT gene and the mechanism by which LAT protects cells from apoptosis is not yet known. It was shown that a miRNA encoded by the HSV-1 LAT gene confers resistance to apoptosis. Mir-LAT downregulates also transforming growth factor (TGF)-beta 1 and SMAD3 expression [80].

### Experimental results and advancements in npcRNAs regulation and expression

Retinoids (Vitamin A) have important functions in development, maintenance of epithelial surfaces, immune competence, and reproduction. All-trans retinoic acid (ATRA) induces the transcription of a selected number of ncRNAs, in a tissue specific way [81]. The ability of ATRA to regulate expression of several hundred genes through binding to nuclear transcription factors is believed to mediate most of these functions. The role of all-trans retinoic may extend beyond the regulation of gene transcription because a large number of noncoding RNAs also are regulated by retinoic acid. Additionally, extra-nuclear mechanisms of action of retinoids are also being identified. Furthermore, specific binding proteins are involved in several of these enzymatic processes as well as in delivery of all-trans retinoic acid to nuclear receptors. Thus, substantial progress has been made in our understanding of retinoid metabolism and function. Retinoids as critical molecules in vision, normal embryonic development, and in control of cellular growth, differentiation, and death throughout life. Retinoids are also therapeutically effective in the treatment of some cancers. It was shown that ATRA induces differentiation of HL60 and NB4 cell lines. The growth-inhibitory effect of all-trans retinoic acid on tumour cells was put in relationship with the expression of retinoic acid receptor alpha gene [82]. Retinoic acid receptors activation triggers transcriptional events leading either to transcriptional activation or repression of retinoid-controlled genes. Using high-density oligonucleotide arrays representing essentially all nonrepetitive sequences on human chromosomes 21 and 22, for mapping the DNA binding sites for Sp1, cMyc, and p53 transcription factors, transcription of non-protein coding RNAs dependent on retinoic acid was shown [83]. It is possible that RAR-α activates many transcription factors as NF-k-B, C/EBP-α, C/EBP-β and C/EBP-ε [84]. In addition, also in HL-60 cells ATRA induces growth arrest. ATRA reduces the expression of Survivin, an anti-apoptotic oncogene, in NB4 cells. ATRA downregulation of RAS correlates with the overexpression of let-7a (Mallardo, manuscript in preparation). miR-15a/miR-16-1 decreases the level of Bcl2: in several human CLL cases, miR-15a and miR-16-1 expression is abolished or diminished. PML-RAR translocation in leukaemia cells is a particular case of malignancies with low aggressive potential, in which therapeutic intervention using retinoic acids has been shown feasible. We speculate that among the ATRA induced genes there are npcRNAs that cooperate to control the transcriptional activity and able to revert the cells into a more differentiated state. We manufactured a DNA array with 500 oligonucleotides relative to human npcRNA genes and monitored their differential expression in NB4 cell line before and after ATRA treatment, individuating 30 npcRNAs that are deregulated by retinoic acid (manuscript in preparation). A functional assignment to these deregulated genes could lead to the identification of candidate tumour modifiers with antiproliferative activity.

### Transcriptional Cancer Therapeutics

The treatment of cancers can be approached either by potentiating established therapies either by intervening on the epigenetic control of gene expression, and also by silencing cancer activator genes. p53, the tumour suppressor and transcriptional shield to cancer, is mutated in many cancers: for some mutations, p53 can be reactivated by peptides and chemical compounds. It was shown that the expression levels of a number of miRNAs is affected by wt-p53, with consequences on the expression of proteins involved in proliferation [85]. p53 is regulated at post-transcriptional level: one of these mechanisms is p53 deacetylation by sirtuins. Cell proliferation activity can be controlled by chemical intervention on histone acetylase and deacetylase enzymes, involved in the regulation of transcriptionally active euchromatin and inactive heterochromatin. Histone Acetyltransferases and Histone Deacetylases (HATs and HDACs) show deregulated activity in many tumours, and treatment of hypermethylated DNA with demethylating agents and drugs as an associated therapy is an approach that can reactivate tumour suppressor expression. The anti-leukemic effects of distinct histone deacetylase (HDAC1 and Sir2) inhibitors, i.e. sodium phenyl butyrate (PB) and vitamin B3, respectively, on human promyelocytic leukaemia cells HL-60, using HDAC Inhibitors alone and in combination with ATRA was recently investigated [86], showing significant acceleration and a high level of granulocytic differentiation. Another level of epigenetic control is achieved by regulation of gene expression by methylation of CpG islands. One of the hallmarks of tumour cells is deregulated DNA methylation patterns. The DNA methyltransferase Dnmt1 is the major mammalian enzyme responsible for maintaining CpG methylation patterns in the cell following replication. Chemical intervention and reactivation of silenced genes is today possible in various cancers. A different approach to induce a change in gene expression is through the uptake inside the cells of RNA-like sequences, with promising therapeutic applications [87,88]. Anti-gene dsRNA, directed to specific regions in gene promoters, can activate and repress gene expression at the DNA level [89], without targeting the chromosomal DNA itself. It is more likely that the agRNA is targeting npc-RNA that is transcribed either in the sense or antisense direction. There is mounting evidence that such antisense transcripts do indeed regulate coding transcripts. agRNAs may elicit or help Argonaute proteins responsible for unwinding of dsRNA during RNAi, to bind a complementary npc-RNA. The complex could recruit proteins or change the conformation of proteins already bound to the chromosomal DNA resulting in gene repression or activation. Over the last years, RNA interference (RNAi) has become a widely used technique that permits the knock-down, and hence functional analysis, of individual genes in vertebrate cells. Experimental steps and expression vectors have been optimised [90] to facilitate the effective knock-down of almost any vertebrate gene product in cultured cells or in experimental animals. Inflammatory pathways, sustained through NF-kB and AKT/PI3K signalling, converge to Stat 3 [91] and Stat1 activation. Activation of Stat1, and its subsequent regulation of clusterin, and STAT3, and its subsequent regulation of c-myc, cyclin D1, Bcl-xL, survivin, VEGF, and miR-21 [92] shows that these pathways sustain cell proliferation and antiapoptotic signalling. STAT3 have been linked with chemoresistance and radioresistance. It is essential to prevent the induction of resistance to taxanes or to other commonly used anticancer drugs, by finding a correct association of therapeutics and modulators of signalling pathways. It is likely that using a combination of different therapeutic compounds and by intervening on various target mechanisms it will be possible to intervene on certain types of cancer through regulation of the transcriptional machinery.

## Conclusion

These data and those on other ncRNAs found implicated in cancer as HULC in hepatocarcinomas [93] and on the involvement of CUDR in drug resistance in squamous carcinoma cell lines [94] show that non-coding RNAs in cancer can be used either as biomarkers and also as therapy targets. It is envisaged the possibility in the future to individuate and act through npcRNAs linked to the transcription machinery in normal cells, either by means of RNA silencing or reactivation of expression, or through chemicals that bind and regulate the activity of npcRNAs.

## Competing interests

The authors declare that they have no competing interests.
